# Development and Reliability of the Basic Skill Assessment Tool for Adolescents with Autism Spectrum Disorder

**DOI:** 10.1155/2017/2403943

**Published:** 2017-01-22

**Authors:** Maethisa Pongsaksri, Suchitporn Lersilp, Sumana Suchart

**Affiliations:** ^1^Department of Occupational Therapy, Faculty of Associated Medical Sciences, Chiang Mai University, Chiang Mai, Thailand; ^2^Rajanagarindra Institute of Child Development, Department of Mental Health, Ministry of Public Health, Chiang Mai, Thailand

## Abstract

The purpose of this study was to improve upon the first version of the basic work skills assessment tool for adolescents with autism spectrum disorder (ASD) and examine interrater and intrarater reliability using Intraclass Correlation Coefficient (ICC). The modified tool includes 2 components: (1) three tasks measuring work abilities and work attitudes and (2) a form to record the number of verbal and nonverbal prompts. 26 participants were selected by purposive sampling and divided into 3 groups—group 1 (10 subjects, aged 11–13 years), group 2 (10, aged 14–16 years), and group 3 (6, aged 17–19 years). The results show that interrater reliabilities of work abilities and work attitudes were high in all groups except that the work attitude in group 1 was moderate. Intrarater reliabilities of work abilities in group 1 and group 2 were high. Group 3 was moderate. Intrarater reliabilities of work attitudes in group 1 and group 3 were high but not in group 2 in which they were moderate. Nevertheless, interrater and intrarater reliabilities in the total scores of all groups were high, which implies that this tool is applicable for adolescents aged 11–19 years with consideration of relevance for each group.

## 1. Introduction

In Thailand, the number of people with autism spectrum disorder (ASD) is approximately 370,000 [1] (Editor, 2015), and the statistic reports of Department of Mental Health have shown that rate of incidence of ASD increased from 4.07 in 2012 to 4.67 in 2013 until being ranked 8th out of other diseases [2].

People with ASD who have an IQ between 50 and 69 can study at regular school and be trained for work or receive vocational training [2]. Most of them learn and remember things from what they see. Some are good at visual/photographic memory. Additionally, their visual-spatial information processing systems are intact. Therefore, they perform tasks using visual-spatial skills very well rather than tasks using verbal skills [3]. They prefer to work with objects or in nonhuman environment [4]. Because they can observe details of things and perceive these precisely [4] and see the relationship of objects and structure them together [3]. They also easily define details and pattern of objects [5]. For this reason, they can perceive patterns of objects and perform copying-the-pattern tasks.

The Department of Mental Health, 2013 [2], has proposed a policy to provide jobs for people with ASD through 3 stages: Choosing, Getting, and Keeping. First of all, the Choosing stage, persons with ASD will be interviewed for what kinds of job may be suitable for them and choose the appropriate jobs for them. Next, the Getting stage, they are trained according to their competence and interest in work under the supervision of job coaches. Finally, the Keeping stage, they will be followed up and supported so that they can continue working and maintain their jobs.

Adolescence is the development period to reduce dependence on their parents [5, 6] and gradually enter into the workplace or higher education [6]. Even though, during adolescence, they still have symptoms of ASD, these may hinder skills necessary for work especially social and communication skills such as poor eye-contact, difficulty to understand what others think or feel, and making mistakes in what to say or do in social situations [5]. Work training benefits their life skill and readiness to work rather than skills acquired from educational institutions [7]. Boonmak, 2010 [8], found that half of the parents are concerned about the future of their children and one-third is afraid that their children cannot be employed. This study recommended providing work for adolescents with ASD.

Work-related skills include basic work skills such as self-advocacy and work behaviors, which are useful for transition to work or vocation [9]. However, in Thailand, there is no measurement tool to assess work performance of adolescents with ASD; hence, the basic work skills assessment tool was developed by Suchart et al., 2015 [10], to examine performance of this group in work. This assessment tool includes 3 task models with different levels of difficulty according to the 3 levels (levels 3, 4, and 5) of* Cognitive Disability Frame of Reference* [11] (Allen, 2013) and the record of work abilities and work attitudes [10]. The record form was developed from the modification of Scale of Independence Behaviors-Revised (SIB-R) in a subscale of work skills with permission [12] and adaptation of the Autism Work Skills Questionnaire, AWSQ [13], Prevocational Checklist of Brown et al., 1977 [14], and self-developed work samples and situational assessment instruments [15].

The basic work skills assessment tool was examined for content validity and interrater and intrarater reliabilities using Intraclass Correlation Coefficient. The 6 subjects were examined and their performances were recorded by the researcher and the other rater who was trained in how to use the new record form, and then the scores from both record forms were computed for interrater reliability. After 2 weeks, the researcher examined the same 6 subjects and recorded their performance again in order to check intrarater reliability. Interrater reliabilities of total scores and scores of work abilities and work attitudes were high, while interrater reliability of those was moderate. The limitations of this study were as follows: the number of subjects was small and most of them were 13 years old; the researcher was also the examiner and the rater, which might bias or influence the other rater. Further research was recommended that a larger number of subjects varying in age should be examined for reliability and the researcher should not be the examiner and the rater [10].

For these reasons, this research was conducted to respond to the recommendation and investigate the reliability of this assessment tool in early, middle, and late adolescents with ASD. Therefore, this assessment tool may be applicable for those in a wider range of adolescence.

## 2. Objectives

The purpose of this study was to improve upon the first version developed by Suchart et al., 2015, and examine interrater and intrarater reliability using Intraclass Correlation Coefficient of the basic work skills assessment tool in early, middle, and late adolescents with ASD.

## 3. Operational Definition

1. Adolescents with ASD refer to persons aged 11 to 19 who have been diagnosed as ASD by medical doctors in Chiang Mai, who are divided into early adolescents aged 11–13 years, middle aged 14–16 years, and late aged 17–19 years.
2. The basic work skills assessment tool refers to an assessment tool developed by Suchart et al., 2015 [10], which is self-criterion-referenced test consisting of the following:
  (2.1) Three task models with different levels of difficulty measuring all items in work abilities and work attitudes.
  (2.2) A record form including 5 items in work abilities and 10 items in work attitudes, on which the number of prompts of the examiner was recorded.
    (2.2.1) Work abilities which refer to cognitive integration necessary for work process consisting of 2 components [16]:
      - Individual competency includes visual discrimination, spatial relations, eye-hand coordination, filling, and sorting [15, 16], which are necessary for basic work.
      - Work components include following verbal instruction or demonstration; copying an example and following correct sequences of work; and maintaining work until finished [12, 15, 16].
    (2.2.2) Work attitudes which refer to appropriate perspectives to work such as seeking help for work tasks when needed, appropriate communication about not understanding the task or procedures, and showing responsibility to complete a task and put away equipment and materials after the completion of the task. Work attitudes require social and communication skills and responsibility in work completion.

The example of this record form is demonstrated as shown in the Appendix.

## 4. Methods

This quantitative study was conducted to further develop and examine the reliability of the basic skill assessment tool and received human ethics approval from the committees of the Faculty of Associated Medical Sciences, Chiang Mai University, before beginning the research process.

## 5. Subjects

Twenty-six subjects were divided into 3 groups. These 3 groups include 10 early adolescents aged 11–13 years, 10 middle adolescents aged 14–16 years, and 6 late adolescents aged 17–19 years and were selected using purposive sampling as per the following inclusion criteria:

1. They could understand instructions, discriminate different colors, and perform tabletop activities for 30 minutes from the reports of their occupational therapists.
2. They could do their own self-care from reports of parents and occupational therapists.
3. They and their parents were willing to participate in the research. The adolescents with ASD signed assent forms and their parents signed consent forms.

In addition, the subjects were excluded from the study if they were color blind, visually impaired, intellectually disable, and adolescents with multiple disabilities.

## 6. Procedure

The procedure is divided into 2 phases.

**Phase 1 .**

It is development through improving the record form and administration according to recommendations from the previous study and the research and ethic committees of the Faculty of Associated Medical Sciences.

This assessment tool was improved as follows:

1. In the record form, verbal and nonverbal behaviors were distinguished to make the prompts clearer and easier for recording.
2. In the steps of administration, the examinees start performing from task model 1: the easiest one, to task model 3: the most difficult one.

**Phase 2 .**

Examination of interrater and intrarater reliabilities of each age group as follows:

1. Select 26 subjects according to the inclusion and divide them into 3 groups: 10 subjects aged 11–13 years, 10 aged 14–16 years, and 6 aged 17–19 years.
2. Prepare the assessment tool consisting of 3 task models and the record form. Each assessment period included one examiner and two raters.
3. Administer the assessment tool in quiet places without any distraction and unfamiliar surroundings to the examinees.
4. The examiners explained and demonstrated the process of assessment which started from task model 1 to task model 3, respectively.
5. If the examinees were able to perform task model 1 without any prompts, then they were provided with task model 2 and if they could perform task model 2 without any prompts, finally, they were provided with task model 3.
6. Two raters observed and recorded the prompts of the examiner.
7. The examiner stopped the assessment after the examinees performed all task models or the examinees were able to perform the task meeting the highest potential of the individual examinee.
8. If the examinees could perform all 3 task models without any prompts, it could be interpreted that this tool was too easy and not appropriate for the examinees.
9. Twenty-six subjects were examined again after 2 weeks and recorded by the one out of two and same rater in step 6 who observed the prompts of the examiner.
10. Calculate interrater reliability of each group of subjects from total scores on the record form of 2 raters using Intraclass Correlation Coefficient (ICC) model 2 [17].
11. The first and second record forms of the same subjects in each group from the rater in item 9 were computed for intrarater reliability using Intraclass Correlation Coefficient model 3 [17].

## 7. Statistic Analysis

Basic demographic data were analyzed by descriptive statistics: frequency mean and standard deviation. Intraclass Correlation Coefficient (ICC) model 2 was used to examine interrater reliability, and ICC model 3 was used to examine intrarater reliability.

## 8. Results

Most subjects aged 11–13 years were males (70%) and had an age range of 11 years and 0 months to 11 years and 11 months (50%). Meanwhile the proportions of male and female subjects aged 14–16 years were equal. In addition, most subjects aged 17–19 years were males (83.30%) and had two age ranges of 17 years and 0 months to 17 years and 11 months and 18 years and 0 months to 18 years and 11 months with equal proportion (see Tables 1–3).

Interrater reliabilities in total scores and work abilities of all groups were high. Nevertheless, interrater reliabilities in work attitudes of groups aged 14–16 and 17–19 years were high (0.832, 0.878 resp.; see Tables 5 and 6), while those of group aged 11–13 years were moderate (.750; see Table 4). Furthermore, intrarater reliabilities in total scores of all groups were high. Moreover, intrarater reliabilities in work abilities of groups aged 11–13 and 14–16 years were high (0.763, 0.900 resp.; see Tables 4 and 5), while the value of group aged 17–19 years was moderate (.690; see Table 6). In addition, intrarater reliabilities in work attitudes of groups aged 11–13 and 17–19 years were high (0.800, 0.800 resp.; see Tables 4 and 6) but that of group aged 14–16 years was moderate (0.700 see Table 5).

## 9. Discussion

When compared with the assessment tool developed by Suchart et al., 2015 [10], interrater reliabilities on the improved assessment tool showed the total scores of all age groups were at the same level as the original one (high). However, intrarater reliabilities on the improved one showed the total scores of all age groups were at a high level, while the original one was at a moderate level. When work abilities and work attitudes were compared between these two versions of the assessment tool, interrater reliabilities of work abilities and work attitudes were also at the same level as the original one (high) except that of the work attitudes of group of 11–13 years. However, intrarater reliabilities of those were at a high level, except that of the work attitudes of age ranges between 14 and 16 years and that of the work abilities of a group aged 17–19 years while those of the previous one were all at the moderate level. The value of intrarater reliability of the work abilities of the group aged 17–19 years and that of the work attitudes of the group aged 14–16 years were 0.690 and 0.700, respectively, which were higher than those of the previous study (0.54 and 0.640, resp.). These results imply that the improved record form with clearer prompts in verbal and nonverbal behaviors and more structured administration which starts from the easiest task model 1 to the most difficult task model 3 make the assessment tool clearer in definition and scoring and administration. More precise definitions and scoring instructions with added clarity [18, 19] with more objective criteria [17] as well as standardized administration [19] make for a robust assessment tool with increased reliability. These results may confirm that the improved assessment tool has greater standardization in measurement, leading to higher reliability.

In the group aged 11–13 years, interrater reliability in work attitudes was moderate (.750). The examiner reported that this group needed a lot of verbal and nonverbal prompts to complete the tasks. In part of the work attitudes session, there are twice the number of items compared with that of work abilities. It may be easier for the raters to miss some observation and record different scores of work attitudes compared to those of work abilities. Similarly, the raters are not sensitive enough in observation and recording the prompts because the sensitivity of behavior observation affects the accuracy of the assessment [20] and may influence reliability. In addition, 70% of this group had stereotypical and repetitive behaviors. These behaviors which associate with sensory processing abnormalities may interfere with social participation and performance in daily life [21] or reduce interaction and participation [22]. Social and communication skills are required in work attitudes; hence this group has a lot of problems in this area and need more prompts than work abilities.

Intrarater reliability of work attitudes in the group aged 11–13 years was still high. That means the behavior items in work attitudes are stable after 2 weeks. It was found that this group could not show appropriate behaviors in work attitudes especially in the items “continued listening while others are speaking” and “keeping eye-contact during conversation” and “showing responsibility to complete a task and put away equipment and materials after the completion of the task.” These findings confirm that items in work attitudes column, under the basic work skills heading (see Appendix) are appropriate to evaluate this age group.

In the group aged 17–19 years, intrarater reliability of work abilities was moderate. All examinees were retested again after 2 weeks which is considered long enough to eliminate a memory effect [17]. Most of adolescents with ASD are good at visual memory, visual-spatial skills, and copying-the-pattern tasks [3, 5]; hence, they can learn this task from the first test due to the influence of learning experience [17]. The lower values of intrarater reliability of work abilities indicate the learning abilities of the oldest age group. This group has more talented learning in visual-spatial skills and visual memory because they also receive occupational therapy continuously for longer time compared to the younger age groups. Therefore, the task models for performing the work abilities may be too easy for this age group.

In the group aged 17–19 years, intrarater reliability of work attitudes was high. There were 6 out of 10 items in work attitudes that they could perform by themselves without any prompts in both the first and the second test. That means these 6 items in work attitudes are too easy for this group. Therefore, after 2 weeks of the retests, their performance did not change much, and the value of intrarater reliabilities of this group was still high.

Intrarater reliabilities of work attitudes in the group aged 14–16 years were moderate which shows fair consistency of the rater or behaviors of this group after 2 weeks. At the retest after 2 weeks, it was reported that 50% of examinees required less prompts. All examinees in this group have been trained in sensory processing activities and social and communication skills in occupational therapy sessions both in schools and in clinics. In addition, adolescents aged 14–16 years need acceptance from friends and have an ability to adjust their behaviors in relationships [23]. Mood and anxiety in an assessment situation can affect their performance [24]. It is possible that they improved these skills before the retests because experiences from these therapy sessions could increase their intrinsic motivation and alleviate their anxiety [21]. Therefore, their performance differed from the first test and the examiner prompted the examinees differently from the first test. This result implies that some behavior items in work attitudes may be simple for them to learn and adjust themselves within 2 weeks.

It was noticed that all 3 groups still need prompts in items, “continued listening while others are speaking”; “keeping eye-contact during conversation”; and “showing responsibility to complete a task and put away equipment and materials after the completion of the task.” All of these work attitudes items require social skills and cognitive competence. Organizing visual-spatial skills with social and communication skills and executive function are necessary to perform work attitudes. Shipman et al., 2011 [25], found that the social related scale of adolescents with ASD aged between 12 and 18 years was lower than the population mean. Furthermore, persons with ASD often have executive function deficits especially cognitive flexibility and planning [26–28]. In addition, they could not retain memory of verbal communication or sound which is immediately lost when compared with the presentation of visual images [5]. This implies that verbal prompts which the examiner often uses in part of work attitudes are quickly lost but the pictures of task models are still on exhibit for them to copy the patterns. In other words, the examiner may need more prompts in part of work attitudes compared to those of work abilities. Therefore, these items in work attitudes are applicable to all age groups due to necessity of prompts. Apart from these, behaviors items needed combination of social communication and cognitive function should be considered to be included in work attitudes.

## 10. Conclusion and Recommendation

Interrater and intrarater reliabilities in the total scores of all groups were high. These results imply that the basic work skills assessment tool is applicable to adolescents aged 11–19 years with the following concerns. To measure adolescents aged 11–13 years, the raters have to be more focused and quickly responsive to the prompts via recording. Moreover, the examiner should ease anxiety and tension of the examinees and inhibit maladaptive behaviors before administration. Furthermore, there should be an example for the examinees to try without scoring for an increase in the understanding of administration. In addition, to retest work abilities of adolescents aged 17–19 years, the examiners have to be concerned with duration and their learning skills. Nevertheless, this assessment tool may be too easy for adolescents with ASD aged 17–19 years. Therefore, for further study, some more difficult task models are being developed and more complex behaviors are being increased in work attitudes. Alternatively, a larger number of adolescents with ASD aged 17–19 years should be utilized to prove the appropriateness of this assessment tool for this group.

## Figures and Tables

**Table 1:** Demographic characteristics of subjects aged 11–13 years.

	Numbers	Percent
*Gender*		
Male	7	70
Female	3	30
Total	10	100.00
*Ages (years)*		
11 years and 0 months to11 years and 11 months	5	50
12 years and 0 months to12 years and 11 months	4	40
13 years and 0 months to13 years and 11 months	1	10
Mean ± SD	12.00 ± 0.56	

**Table 2:** Demographic characteristics of subjects aged 14–16 years.

	Numbers	Percent
*Gender*		
Male	5	50
Female	5	50
Total	10	100.00
*Ages (years)*		
14 years and 0 months to14 years and 11 months	5	50
15 years and 0 months to15 years and 11 months	3	30
16 years and 0 months to16 years and 11 months	2	20
Mean ± SD	14.92 ± 0.75	

**Table 3:** Demographic characteristics of subjects aged 17–19 years.

	Numbers	Percent
*Gender*		
Male	5	83.30
Female	1	16.70
Total	6	100.00
*Ages (years)*		
17 years and 0 months to17 years and 11 months	3	30
18 years and 0 months to18 years and 11 months	0	0
18 years and 0 months to18 years and 11 months	3	30
Mean ± SD	18.54 ± 1.01	

**Table 4:** Interrater and intrarater reliabilities of work abilities, work attitudes, and total using Intraclass Correlation Coefficient (ICC) of subjects aged 11–13 years. The table shows interrater reliability using ICC which ranged from .750 to .934 and intrarater reliability using ICC which ranged from .763 to .800 (*p* < .05).

Items	Interrater reliability ICC	Intrarater reliability ICC
Work abilities	.962	.763
Work attitudes	.750	.800

Total	.934	.800

**Table 5:** Interrater and intrarater reliabilities of work abilities, work attitudes, and total using Intraclass Correlation Coefficient (ICC) of subjects aged 14–16 years. The table shows interrater reliability using ICC which ranged from .832 to .974 and intrarater reliability using ICC which ranged from .700 to .919 (*p* < .05).

Items	Interrater reliability ICC	Intrarater reliability ICC
Work abilities	.965	.900
Work attitudes	.832	.700

Total	.974	.919

**Table 6:** Interrater and intrarater reliabilities of work abilities, work attitudes, and total using Intraclass Correlation Coefficient (ICC) of subjects aged 17–19 years. The table shows Interrater reliability using ICC which ranged from .878 to .997 and intrarater reliability using ICC which ranged from .690 to .852 (*p* < .05).

Items	Interrater reliability ICC	Intrarater reliability ICC
Work abilities	.985	.690
Work attitudes	.878	.800

Total	.997	.852

## Appendix

### A Record Form of Basic Work Skills for Adolescents with ASD

*Basic Work Skills*

1. Work abilities
  (1.1) following verbal instruction or demonstration
  *Numbers of prompts*
      Non-verbal: —
      Verbal: —
  *Types of prompts*: —
  (1.2) sorting, filling and copying according to the task model with correct sequences and tidy organization
  *Numbers of prompts*
      Non-verbal: —
      Verbal: —
  *Types of prompts*: —
  (1.3) maintaining work until finished
  *Numbers of prompts*
      Non-verbal: —
      Verbal: —
  *Types of prompts*: —
  *Numbers of prompts*
      Non-verbal: —
      Verbal: —
  *Types of prompts*: —
  *Numbers of prompts*
      Non-verbal: —
      Verbal: —
  *Types of prompts*: —
2. Work attitudes
  (2.1) introducing yourself
  *Numbers of prompts*
      Non-verbal: —
      Verbal: —
  *Types of prompts*: —
  (2.2) responding to greeting appropriately
  *Numbers of prompts*
      Non-verbal: —
      Verbal: —
  *Types of prompts*: —
  (2.3) keeping eye-contact during conversation
  *Numbers of prompts*
      Non-verbal: —
      Verbal: —
  *Types of prompts*: —
  (2.4) continued listening while others are speaking
  *Numbers of prompts*
      Non-verbal: —
      Verbal: —
  *Types of prompts*: —
  *Numbers of prompts*
      Non-verbal: —
      Verbal: —
  *Types of prompts*: —
  *Numbers of prompts*
      Non-verbal: —
      Verbal: —
  *Types of prompts*: —
  (2.5) seeking help for work tasks when needed
  *Numbers of prompts*
      Non-verbal: —
      Verbal: —
  *Types of prompts*: —
  *Numbers of prompts*
      Non-verbal: —
      Verbal: —
  *Types of prompts*: —
  (2.6) appropriate communication about not-understanding the task or procedures
  *Numbers of prompts*
      Non-verbal: —
      Verbal: —
  *Types of prompts*: —
  (2.7) showing responsibility to complete a task and put away equipment and materials after the completion of the task
  *Numbers of prompts*
      Non-verbal: —
      Verbal: —
  *Types of prompts*: —
